# Molecular Variation in Porcine Circovirus Type 2 in Jalisco, Mexico, and Its Potential Impact on Vaccine Efficacy

**DOI:** 10.3390/vaccines14070564

**Published:** 2026-06-26

**Authors:** Alberto Jorge Galindo-Barboza, José Francisco Rivera-Benítez, Jazmín De la Luz-Armendáriz, José Iván Sánchez-Betancourt, Jesús Hernández, Alexel Jesús Burgara-Estrella, Suzel Guadalupe Sauceda-Cerecer, Laura Márquez-Valdelamar, Jaime Enrique De Alba-Campos

**Affiliations:** 1 Programa de Doctorado en Ciencias de la Producción y de la Salud Animal, Universidad Nacional Autónoma de México, UNAM, Mexico City 04510, Ciudad de México, Mexico; aljogaba@gmail.com; 2 Laboratorio de Virología, Centro Nacional de Investigación Disciplinaria en Salud Animal e Inocuidad, Instituto Nacional de Investigaciones Forestales, Agrícolas y Pecuarias, INIFAP, Mexico City 05110, Ciudad de México, Mexico; 3 Facultad de Medicina Veterinaria y Zootecnia, Universidad Nacional Autónoma de México, UNAM, Mexico City 04510, Ciudad de México, Mexico; delaluzarmendarizj@fmvz.unam.mx (J.D.l.L.-A.); ivan.sanchez@posgrado.unam.mx (J.I.S.-B.); 4 Laboratorio de Inmunología, Centro de Investigación en Alimentación y Desarrollo, Asociación Civil., Hermosillo 83304, Sonora, Mexico; jhdez@ciad.mx; 5 Micro y Nano Tecnologías Biomédicas, Departamento de Investigación en Física, Universidad de Sonora, Hermosillo 83000, Sonora, Mexico; alexel.burgara@unison.mx; 6 Grupo Estatal de Vigilancia Epidemiológica, Comité Estatal para el Fomento y Protección Pecuaria del Estado de Jalisco, Sociedad Civil, El Salto 45690, Jalisco, Mexico; suzelsauceda@gmail.com; 7 Laboratorio de Secuenciación Genómica de la Biodiversidad y de la Salud, Universidad Nacional Autónoma de México, UNAM, Mexico City 04510, Ciudad de México, Mexico; lmarquez@ib.unam.mx; 8 Unión Regional de Porcicultores de Jalisco, El Salto 45680, Jalisco, Mexico; mvzjeac@hotmail.com

**Keywords:** porcine circovirus-associated diseases, PCV2, farms, serum, PCR

## Abstract

**Background/Objectives:** Porcine circovirus type 2 (PCV2) remains a major viral agent in pig production worldwide due to its association with economically relevant diseases and productivity losses. Nine genotypes (PCV2a–PCV2i) have been reported, with successive genotype shifts characterized by the historical predominance of PCV2a, the expansion of PCV2b, and the emergence of PCV2d as the predominant genotype in several swine-producing countries. The aim of this study was to characterize the ORF2 gene of PCV2 circulating in Jalisco, Mexico, to provide updated information for regional surveillance and control strategies. **Methods:** Samples were collected from 80 pig farms located in four regions of Jalisco with different pig density levels and production systems. PCV2-positive samples were subjected to ORF2 amplification and sequencing. Genotype assignment, phylogenetic analysis, and in silico recombination screening using multiple detection methods were performed. All sequences were deposited in GenBank. **Results:** A total of 70 ORF2-PCV2 sequences were obtained and assigned to two genotypes: PCV2d (51/70, 72.9%) and PCV2a (19/70, 27.1%). The sequences were submitted to GenBank under accession numbers PV235521–PV235590. Recombination analysis identified seven recombinant sequences, and unusual ORF2 extensions were detected in some sequences, evidencing the presence of genetic variants circulating in the region. **Conclusions:** These findings confirm the predominance of PCV2d in Jalisco while highlighting the continued circulation of PCV2a. The coexistence of both genotypes, together with recombinant sequences and ORF2 extensions, indicates ongoing PCV2 genetic diversification in the region. Continuous molecular surveillance remains essential to monitor viral evolution, support genotype-informed control strategies, and strengthen swine health programs.

## 1. Introduction

Porcine circovirus type 2 (PCV2) belongs to the genus *Circovirus* within the family *Circoviridae*. It is a small, non-enveloped virus containing a circular single-stranded DNA genome of approximately 1.7 kb. To date, nine PCV2 genotypes, designated PCV2a to PCV2i, have been described worldwide. The genotype distribution of PCV2 has changed over time: PCV2a was initially the most common genotype but was surpassed by PCV2b following its expansion in several regions, including Mexico [1,2,3]. PCV2c was first reported in Denmark in 2008 and subsequently identified in Brazil and China [4,5,6], while PCV2d, which emerged around 2000, has progressively increased in frequency and is now commonly found in clinical cases from major swine-producing regions of Asia, Europe, and the Americas [7,8,9,10,11].

Transmission of PCV2 occurs primarily through contact with infected animals, although indirect exposure through contaminated fomites can also contribute to viral spread [12]. Its high resistance to environmental and chemical factors [13] enables prolonged persistence in pig production systems, favoring repeated exposure among animals. This persistence, together with the virus’s tropism for immune cells, leads to immunosuppression and co-infections [14].

PCV2 infection can remain subclinical, but it may also be associated with different disease presentations grouped under the term porcine circovirus-associated diseases (PCVADs). These include post-weaning multisystemic wasting syndrome (PMWS), porcine dermatitis and nephropathy syndrome (PDNS), porcine respiratory disease complex (PRDC), and reproductive failure. Although PDNS has been epidemiologically linked to PCV2 infection, its direct causality has not been fully demonstrated [15]. Because PCV2 affects multiple productive and health outcomes, it remains a pathogen of high economic relevance for the swine industry. Its control depends on integrated herd-level strategies that feature a combination of vaccination [16], biosecurity, and management practices aimed at reducing stressors and supporting overall herd health [15]. In addition, continuous molecular surveillance is essential, as the virus has undergone successive genotype shifts over time—from PCV2a to 2b, 2c, and more recently 2d—altering its prevalence and distribution across regions.

PCV2 diversity results from the combined effects of viral, host, and production-related factors. Mutations can accumulate in its DNA genome over time, generating nucleotide and amino acid variation among circulating strains [17]. In addition, immune responses at the herd level, including those induced by natural infection or vaccination, may contribute to the selection of variants; however, the magnitude and direction of this effect depend on the epidemiological context and require specific evaluation [18]. The wide distribution of PCV2 across swine-producing regions also increases the chance of co-circulation of different genotypes, which can facilitate recombination events [10]. Farm management, biosecurity practices, animal movement, and production density may further influence viral persistence and transmission dynamics [19,20]. Considering these factors together is important for interpreting PCV2 evolution and for designing surveillance and control strategies adapted to regional conditions.

In the Mexican context, comprehensive data on the frequency of PCV2 infection are still scarce; however, recent reports have documented positivity rates of 91.67% in farms in the northwest region of the country [21] and 75% in Jalisco [19]. Although the frequency of infection appears to be high, information regarding the genetic variability of PCV2 remains limited, largely because few epidemiological studies have been conducted at the regional or state level. Moreover, most available reports are restricted to clinical case descriptions in specific production units [22] or diagnostic surveys based solely on serology [21,23], and the few molecular analyses that exist are based on data generated several years ago in different states and mainly report the PCV2a and PCV2b genotypes [3,24,25]. The swine industry in Jalisco comprises a wide range of production systems, from technologically intensive commercial farms with export capacity to small-scale family operations on backyard units, reflecting a structurally heterogeneous sector [26]. This diversity poses significant challenges for the implementation of standardized disease control strategies and may contribute to the co-circulation and maintenance of multiple viral variants within the region. Therefore, in this study, we aim to characterize the ORF2 of PCV2 strains circulating in Jalisco, Mexico, to generate updated data that can support the development of control and epidemiological surveillance strategies tailored to regional needs.

## 2. Materials and Methods

### 2.1. Research Site and Samples

This study was carried out in Jalisco, Mexico, using samples from 80 commercial pig farms located in four regions with different pig-density levels [26]. Farms were assigned to regional clusters and classified by size as either semi-intensive units, with 21–500 breeding sows, or intensive units, with ≥500 breeding sows. They were also categorized according to production system as farrow-to-finish (FF) or multisite (MS) farms. On each farm, 60 pigs were sampled to cover the main production stages, covering suckling pigs, weaning pigs, growing pigs, finishing pigs, and pregnant sows. Sampling was performed between January and March 2023 as part of a cross-sectional survey of commercial pig farms in Jalisco.

Blood was obtained from the cranial vena cava and collected in sterile 5 mL tubes without anticoagulant. Samples were left at room temperature for approximately 30 min to allow for clot formation and then centrifuged at 1500× *g* for 10 min to separate the serum. The serum fraction was aliquoted into labeled microtubes and stored at −20 °C until processed. Serum samples were pooled according to the production stage, yielding up to 12 pools per farm. In multisite systems, where not all production stages were practiced due to farm structure or management practices, only six pools were obtained. Overall, 4207 pigs were sampled, resulting in a total of 844 pools. Each pool contained serum from up to five pigs from the same production stage. Pooling was used as an epidemiological screening strategy at the pool level, rather than for individual-level diagnosis. To reduce the potential dilution effect associated with pooling, the pool size was limited to five sera, and only qPCR-positive pools with Ct values ≤ 30 were selected for ORF2 amplification and sequencing. All laboratory procedures were performed at the Virology Laboratory of CENID SAI-INIFAP (Palo Alto, Mexico City, Mexico).

### 2.2. Molecular Detection and ORF2 Characterization

DNA was obtained from pooled serum samples using the QIAwave^®^ DNA Blood & Tissue Kit (QIAGEN, Hilden, Germany, Cat. No. 69556), according to the manufacturer’s instructions, and stored at −76 °C until use. PCV2 detection was performed via quantitative PCR (qPCR) targeting the ORF2 gene with the QuantiTect^®^ Probe PCR Kit (QIAGEN, Hilden, Germany, Cat. No. 204345), following the protocol described by Olvera et al. (2007) [27]. Reactions were carried out in a final volume of 10 μL, consisting of 5 μL of 2× QuantiTect Probe PCR Master Mix, 2 μL of DNA template (≤500 ng/reaction), 1.75 μL of primer–probe mix, and 1.25 μL of RNase-free water. Final primer and probe concentrations were 1.2 μM and 0.2 μM, respectively. Pools with Ct values ≤ 30 were considered positive and selected for ORF2 sequencing.

The ORF2 gene was amplified by endpoint PCR using primers previously described by De La Luz-Armendáriz et al. [24], which target a 765 bp fragment, together with the GoTaq Green^®^ PCR Kit (Promega Corporation, Madison, WI, USA, Cat. No. M7122). Each 12.5 µL reaction contained 6.25 µL of Master Mix, 2 µL of primer mix, 2.5 µL of DNA template, and 1.75 µL of RNase-free water. Amplification was carried out with an initial denaturation step at 95 °C for 2 min, followed by 40 cycles of denaturation at 95 °C for 30 s, annealing at 56 °C for 1 min, and extension at 72 °C for 1 min. A final extension was performed at 72 °C for 5 min. PCR products were combined with GelPilot^®^ DNA Loading Dye (QIAGEN, Hilden, Germany, Cat. No. 239901) and resolved on 1.5% agarose gels prepared in 1× TAE buffer at 90 V for approximately 50 min. Bands were stained with ethidium bromide (EtBr) and visualized under UV light. The expected amplicon size was verified using a 1 Kb Plus DNA Ladder (Invitrogen™, Thermo Fisher Scientific Baltics UAB, Vilnius, Lithuania, Cat. No. 10787026).

Amplicons with positive PCR results were purified using the QIAquick^®^ PCR Purification Kit (QIAGEN, Hilden, Germany, Cat. No. 28106) according to the manufacturer’s instructions and eluted in 80 µL of elution buffer. DNA quantity was assessed by agarose gel electrophoresis through comparison with bands from a DNA ladder of known concentration. Amplicons with sufficient band intensity were selected for sequencing. Sanger sequencing was carried out at the Genomic Sequencing Laboratory of the Biodiversity and Health Institute, Instituto Nacional de la Biodiversidad (INB), Universidad Nacional Autónoma de México (UNAM), following standard sequencing procedures. The resulting chromatogram files were used for sequence assembly, and the final sequences were deposited in GenBank. During sequence processing, the 5′ and 3′ untranslated regions were trimmed, retaining only the 705 bp ORF2 coding region for downstream analyses. Assembly and editing were performed using MEGA 12 software [28].

### 2.3. Phylogenetic Analysis and Genotype Classification

Phylogenetic relationships among the ORF2 sequences generated in this study were evaluated using reference sequences obtained from GenBank and from previously published genotype classification datasets [17]. These reference sequences were included as anchors to support genotype assignment during alignment and tree interpretation.

Sequences were aligned in MEGA version 12 using the Clustal W algorithm [29]. Phylogenetic reconstruction was performed with the Neighbor-Joining method [30], and genetic distances were calculated under the Tamura–Nei model [31] as base substitutions per site. A gamma distribution with a shape parameter of 0.58 was used to model among-site rate variation. Sites containing gaps or missing data were removed using the complete deletion option. Branch support was evaluated with 1000 bootstrap replicates, and bootstrap values were displayed on the relevant nodes to assess cluster reliability [32].

### 2.4. Estimation of Sequence Divergence

To explore the genetic variability of PCV2, pairwise sequence divergence was assessed at both nucleotide and amino acid levels. Nucleotide distances were estimated using the Tamura–Nei model, while amino acid divergence was calculated using the Jones–Taylor–Thornton (JTT) model [33], selected based on the best fit under a maximum likelihood approach. Ambiguous positions were removed using pairwise deletion for amino acid comparisons. All analyses were conducted in MEGA version 12 and the R language, employing the ape [34] and pheatmap [35] packages for distance matrix computation and graphical representation. The amino acid table was designed with the Jalview platform [36].

### 2.5. Epitope Identification

B-cell epitopes within the ORF2 amino acid sequences were predicted using the BepiPred-2.0 tool [37,38], available at http://tools.iedb.org/main/bcell/ (accessed on 20 March 2026). The predicted epitope regions were subsequently analyzed in MEGA version 12 to assess sequence variability and visualized using the pheatmap package version 1.0.13 in R version 4.6.1. Conserved and variable regions were identified across all sequences, with a particular focus on epitope sites to detect potential amino acid changes that could affect antigenic properties.

### 2.6. Recombinant Identification

Potential recombination events within the ORF2 amino acid sequences were investigated using the RDP5 Beta 5.93 software package [39]. Multiple detection methods were employed during analysis, including RDP [40], GENECONV [41], BootScan [42], MaxChi [43], Chimaera [44], SiScan [45], and 3Seq [46], with default parameters. Only recombination events supported by at least four different methods and with Bonferroni-corrected *p*-values < 0.05 were considered reliable.

All analyses were conducted using standardized protocols and validated software to ensure accuracy and reproducibility. The results obtained through these methodologies provided a comprehensive overview of the genetic characteristics of the PCV2 strains analyzed, as detailed in the following section. Additionally, sequences showing recombination signals (recombinant sequences) and their putative parental strains were mapped using QGIS to assess their biological plausibility in relation to the geographical characteristics of the region.

## 3. Results

### 3.1. Molecular Analysis

A total of 235 pools were positive, and 112 of them showed a Ct value below 30; of these, 70 ORF2-PCV2 samples were successfully purified, sequenced, and assembled. The Ct values of these 70 sequenced samples ranged from 18.08 to 30.00, with a mean of 27.37 and a median of 28.03. These sequences corresponded to 51 PCV2d (72.9%) and 19 PCV2a (27.1%); all 70 sequences generated in this study were deposited in GenBank under accession numbers PV235521–PV235590. Table 1 lists the obtained sequences along with detailed information on their geographic and/or epidemiological origin, Ct value, and genotype.

In addition to the 70 ORF2 sequences with the typical length, two additional sequences showed an extended ORF2 due to the presence of 24 extra nucleotides, resulting in a predicted capsid protein of 238 amino acids. These two extended ORF2 variants corresponded to one PCV2a and one PCV2d sequence. Because they differed in length from the main ORF2 dataset and are currently being characterized as part of a whole-genome analysis, they were analyzed separately and were not merged with the 70 sequences used for comparative analyses in order to avoid alignment bias. This observation is further discussed due to its potential biological relevance.

### 3.2. Phylogenetic Analysis

**Figure 1:**
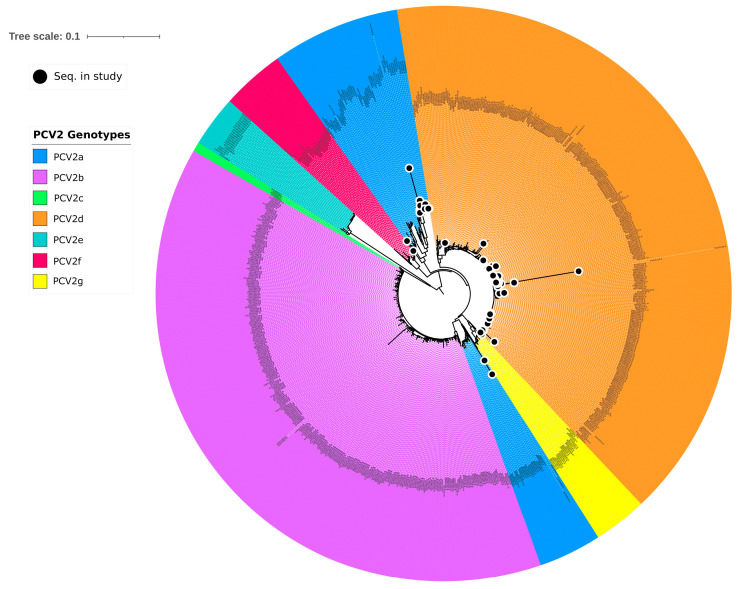
Neighbor-Joining tree constructed from PCV2 ORF2 nucleotide sequences from Jalisco, Mexico. The analysis included the 70 sequences generated in this study and 764 reference sequences used as anchors for the recognized PCV2 genotypes. Genetic distances were calculated with the Tamura–Nei model, applying a gamma distribution (shape parameter = 0.58) to account for among-site rate variation. After removing positions with gaps or missing data by complete deletion, the final dataset contained 705 nucleotide sites. Sequences from this study are indicated with black circles. The tree was annotated and displayed using the Interactive Tree Of Life (iTOL) platform.

A total of 70 nucleotide sequences were included in the analysis, resulting in a final alignment of 705 positions (GenBank Accessions PV235521 to PV235590). The resulting phylogenetic tree (Figure 1) displays optimal topology and clearly differentiates the main PCV2 genotypes. Black circles indicate the sequences generated in this study, highlighting the presence of PCV2a and PCV2d among the samples analyzed.

**Figure 2:**
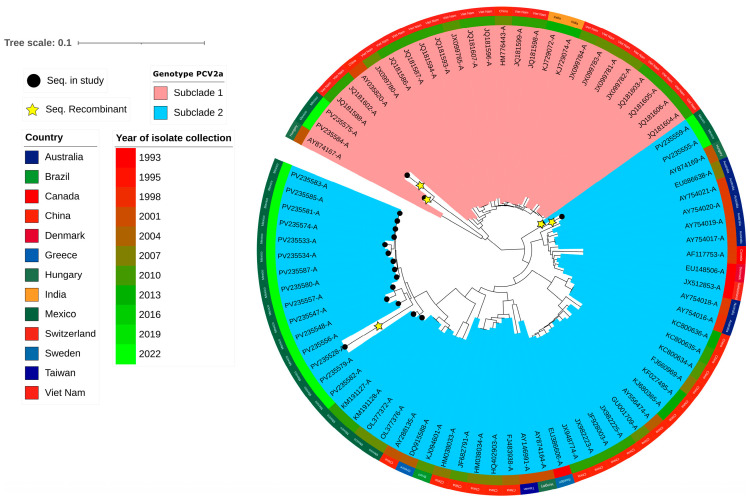
Neighbor-Joining tree showing the phylogenetic placement of the 19 PCV2a sequences generated in this study together with 63 PCV2a reference sequences. Two supported groups, Subclade 1 and Subclade 2, are indicated in the tree. Sequence names include the country and year of isolation. Black dots denote sequences from this study, and stars denote sequences with recombination signals. The mean divergence between both subclades was 11.3%, estimated in R from cophenetic distances obtained from the tree topology using the ape and phytools packages. The tree was visualized with iTOL v6.

Figure 2 displays the 82 sequences used to contextualize PCV2a, including the 19 PCV2a sequences generated in this study and 63 PCV2a reference (anchor) sequences. Although the full dataset was used to infer the phylogenetic topology, the two clades resolved within PCV2a are primarily defined by the 19 sequences obtained in this study. Of these, 2 grouped within Subclade 1 and 17 within Subclade 2; the remaining sequences in each subclade correspond to reference strains included only for phylogenetic anchoring. The average genetic distance between both subclades was 0.1133 (11.3%), calculated as the mean cophenetic distance between all pairwise combinations of sequences from each subclade based on the phylogenetic tree topology. All comparative and distance-based analyses for PCV2a were performed exclusively on the 19 sequences generated in this study. The figure shows the country of origin and collection year for each of the 82 sequences. Black dots indicate sequences generated in this study, and stars indicate sequences with recombination signals. These patterns are further discussed below.

### 3.3. Sequence Divergence Analysis

The pairwise nucleotide distances among the 70 PCV2 ORF2 sequences revealed substantial genetic divergence, ranging from 0.00 to 0.38 substitutions per site. However, amino acid divergence was considerably lower, suggesting that the capsid protein remains relatively conserved despite nucleotide variation.

**Figure 3:**
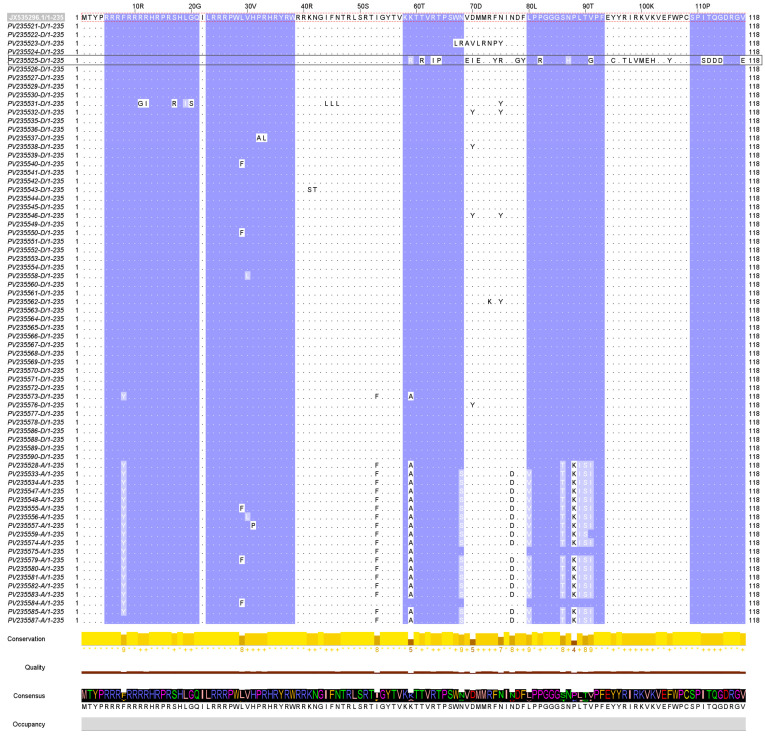

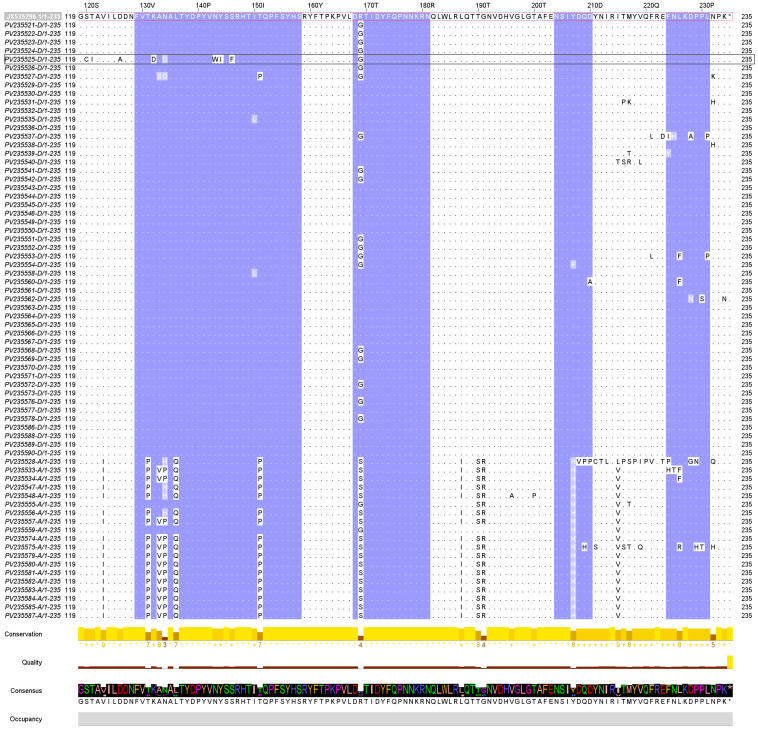
Amino acid alignment of PCV2 ORF2 sequences generated in this study, using JX535296.1 (PCV2d) as the reference strain. Conserved and variable amino acid positions are displayed across the 70 sequences, and predicted antigenic epitopes are shaded in purple. PV235525, which showed greater amino acid divergence within the PCV2d group, is highlighted with a box. The alignment was visualized in Jalview version 2.11.5.1. The asterisk (*) denotes the stop codon at the end of the translated ORF2 sequence. Letters indicate amino acid residues at variable positions using the standard one-letter amino acid code, whereas dots indicate positions identical to the reference sequence.

The amino acid alignment matrix (Figure 3), generated using Jalview [36] and the reference sequence JX535296.1 (PCV2d), contains conserved and variable positions across all 70 sequences. Epitopes predicted in the capsid protein are shaded in purple within the alignment and correspond to the regions summarized in Table 2. The observed amino acid variation patterns were consistent with the previously established genotype classification: sequences identified as PCV2a and PCV2d formed clearly distinguishable groups, characterized by higher intra-genotype similarity and greater divergence between genotypes. Notably, within the PCV2d group, sequence PV235525 exhibited a higher number of amino acid differences compared to other PCV2d sequences, making it visibly distinct within the alignment. However, this sequence was obtained from a sample taken from a seemingly healthy pig. These findings support the presence of a clear genetic structure and heterogeneity among the PCV2 strains circulating in the studied region.

### 3.4. Identified Epitopes

To assess epitope variability, the 70 PCV2 ORF2 sequences obtained from swine farms in Jalisco were compared against a reference sequence from the USA, classified as PCV2d (GenBank accession JX535296.1) [47]. This reference was selected because it corresponds to the first reported case of PCV2d in the Americas and has been associated with increased pathogenicity. Based on this sequence, linear B-cell epitopes were predicted using the BepiPred-2.0 tool, resulting in nine potential targets within the ORF2 amino acid sequence. These predicted epitopes are summarized in Table 2.

**Table 2:** Linear B-cell epitopes predicted in the PCV2 ORF2 amino acid sequence using BepiPred-2.0 with the Random Forest model. For each epitope, the table lists the amino acid and nucleotide positions, peptide sequence, peptide length, and mean BepiPred score with standard deviations. Amino acid residues with scores above the 0.5 threshold were considered potentially antigenic. Predictions were generated using the PCV2d reference sequence JX535296.1. Epitope 7 (Ep7), previously identified and experimentally validated [48], is included because of its immunological relevance. These predictions are computational and should be interpreted as guidance for experimental validation rather than as substitutes for serological assays.

Epitope	Position aa	Position nt	Peptide	Length aa	Score
Ep1	5–21	13–63	RRRFRRRRHRPRSHLGQ	17	0.6001
Ep2	23–38	67–114	LRRRPWLVHPRHRYRW	16	0.5134
Ep3	58–68	172–204	KKTTVRTPSWN	11	0.5854
Ep4	80–93	238–279	LPPGGGSNPLTVPF	14	0.5818
Ep5	109–118	325–325	SPITQGDRGV	10	0.5507
Ep6	129–158	385–474	FVTKANALTYDPYVNYSSRHTITQPFSYHS	30	0.5596
Ep7	168–181	502–543	DRTIDYFQPNNKRN	14	0.5792
Ep8	204–210	610–630	NSIYDQD	7	0.5360
Ep9	224–231	670–693	FNLKDPPL	8	0.5690

aa: amino acid, nt: nucleotide.

Analysis of the nine predicted B-cell epitopes showed that variability was best captured by the number of sequences carrying at least one amino acid change, rather than by the percentage of divergence per site. Under this criterion, Ep7 emerged as the most variable region, displaying low per-site divergence but the highest frequency of mutated sequences across the dataset, making it the epitope with the largest qualitative diversity. Ep4 also showed high variability, though largely restricted to PCV2a, while Ep6 and Ep3 exhibited moderate to high variability, distributed across several sequences. In contrast, Ep1, Ep2, and Ep5 remained largely conserved, with only isolated changes, and Ep8 and Ep9 were mostly conserved in PCV2d but showed sporadic variability in PCV2a. Overall, these patterns confirm that Ep7 is the principal hotspot of amino acid variation, not because of the magnitude of its changes but due to the high frequency of distinct variants found among circulating strains.

**Figure 4:**
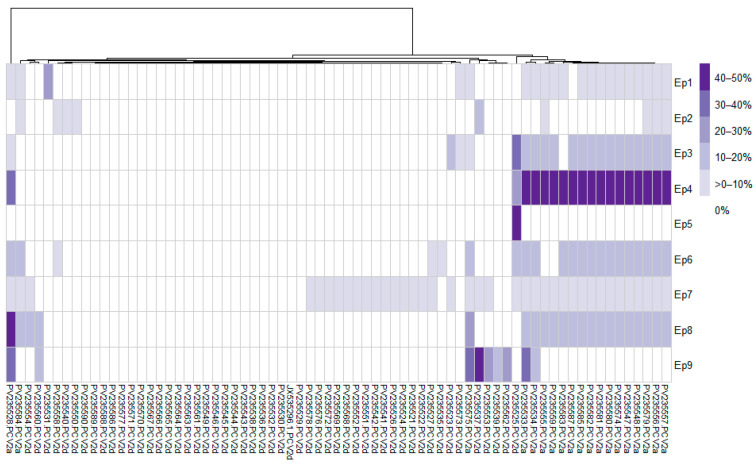
Heatmap depicting the variability of predicted B-cell epitopes across 70 PCV2 ORF2 sequences from Jalisco, Mexico, in comparison with one highly pathogenic reference sequence (GenBank JX535296.1, PCV2d, USA). Epitope-specific amino acid divergence (%) was calculated relative to this reference. Lighter shades represent higher conservation, while darker tones indicate greater variability. Sequences are hierarchically clustered based on epitope-specific amino acid divergence relative to the reference, and the dendrogram above the heatmap indicates groups of sequences with higher similarity. Some sequences from other genotypes (e.g., PCV2a) may appear interspersed if their epitope regions show relatively high similarity to the reference.

A heatmap was generated to visualize amino acid variation within each of the nine predicted B-cell epitopes (Figure 4). Columns were hierarchically clustered to reveal patterns of similarity. The color gradient reflects the degree of divergence, with lighter tones indicating greater conservation and darker shades indicating higher variability. The heatmap shows that Ep1, Ep2, and Ep5 were largely conserved across the analyzed sequences, whereas Ep4 displayed marked variability, mainly among PCV2a sequences. Ep6, Ep8, and Ep9 showed intermediate or sporadic variability, while Ep7 showed changes across multiple sequences, consistent with the frequency-based variability analysis described above.

### 3.5. Analysis of Recombinant Sequences

Seven recombinant sequences were identified through in silico recombination analysis using multiple detection methods. The putative recombinants showed strong statistical support, with *p*-values < 0.001 in most detection methods. Breakpoints were consistently located within the ORF2 coding region. Detailed information on the recombinant sequences, parental strains, breakpoint positions, and statistical support from each method is presented in Table 3.

**Figure 5:**
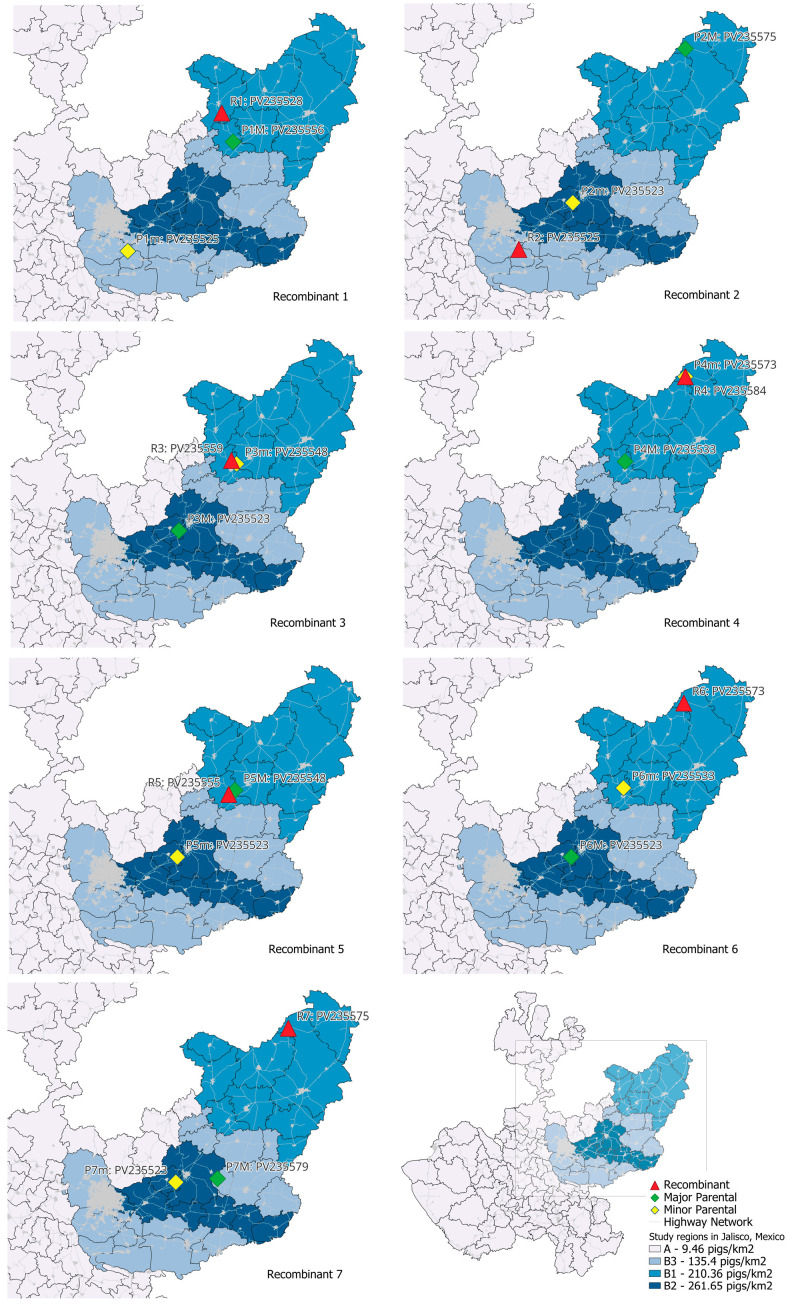
Geographic distribution of recombinant PCV2 sequences and their putative parental strains identified in Jalisco, Mexico. Each panel corresponds to one recombinant event (Recombinants 1–7). Red triangles indicate recombinant sequences, green diamonds indicate major parental sequences, and yellow diamonds indicate minor parental sequences. Gray lines represent the main transportation corridors for animals, supplies, and technical personnel connecting the regions with the highest swine density, including the Guadalajara metropolitan area, Altos Norte, and Altos Sur (B1, B2, and B3 regions). The inset map shows the location of the study area within Jalisco, Mexico.

Figure 5 illustrates the geographic distribution of the recombinant sequences identified in the state of Jalisco, clustered along a swine production corridor linking the Guadalajara metropolitan area with the Altos Norte and Altos Sur regions (B1, B2 and B3 Regions).

## 4. Discussion

Globally, PCV2 diversity is mainly characterized by four widely reported genotypes: PCV2a, PCV2b, PCV2c, and PCV2d. Changes in genotype predominance over time have been linked to viral dissemination through trade routes, animal movement, and modifications in swine production systems [49]. In this context, the observed predominance of PCV2d in Jalisco is consistent with the genotype displacement reported in other regions, where PCV2d has increased in frequency while earlier genotypes, particularly PCV2a and PCV2b, have become less prevalent. This dynamic has been documented in multiple regions worldwide and has been associated with PCV2d’s reported biological features, including its higher viral load, greater genetic diversity, and possible antigenic differences compared with vaccines based on PCV2a [7,47]. Consistent with these findings, studies conducted in the United States between 2014 and 2016 found that the PCV2d-2 subgenotype was most frequently detected in the lung tissues of pigs, despite high vaccination coverage [11]. Within the region considered in this study, some producers perceive PCV2 vaccination as insufficient to prevent clinical problems; however, this perception is subjective and requires validation through controlled field studies including vaccination protocols, immune response, and clinical outcomes.

The finding that approximately 27% of the analyzed sequences correspond to PCV2a indicates the coexistence of at least two circulating genotypes in the region. The observed nucleotide divergence, which reached up to 0.38 substitutions per site, may be attributed to the coexistence of distinct genotypes (PCV2a and PCV2d) within the dataset. This may reflect the persistence of ancestral strains, together with the recent expansion of PCV2d in a production environment where PCV2 vaccination is commonly reported. However, the role of vaccination in shaping this genotype distribution cannot be directly assessed without detailed information on vaccine type, schedule, coverage, and immune response. Of the 70 sequences obtained in this study, three originated from farms where PCV2 vaccination was not used; all three sequences were classified as PCV2d. Before 2018, PCV2b was the dominant genotype in different regions of the country (87.5%), followed by PCV2a [2]. However, more recent studies have reported a higher frequency of PCV2d, reflecting an epidemiological pattern consistent with the present study area and other areas in central-western Mexico [24].

The phylogenetic analysis revealed that the PCV2a sequences in this study clustered into two well-defined subclades, despite belonging to the same genotype. This separation was supported by a bootstrap value >70 and by the presence of long branches within each group, indicating considerable evolutionary divergence. The average genetic distance between the two subclades was 0.1133 substitutions per site, below the 13% intra-genotype threshold proposed for PCV2 genotype definition but sufficiently high to indicate evolutionarily distinct groupings within PCV2a [17].

This divergence may be explained by geographical factors associated with the reference sequences used, such as region-specific distribution—e.g., one subclade predominantly comprising sequences from Asia and the other from the Americas—or by temporal differences, reflecting the coexistence of older and more recent strains. It may also reflect parallel evolution of independent variants within the same genotype among the sequences generated in this study, consistent with a process of intra-genotypic diversification.

The identification of this phylogenetic structure within PCV2a highlights the evolutionary complexity of the virus and may have important implications for epidemiological surveillance and vaccine design, especially if these subclades exhibit antigenic variation or distinct circulation patterns. These findings underscore the importance of continued monitoring of PCV2 evolution, even within genotypes that have traditionally been considered homogeneous.

The predominance of PCV2d observed in this study matches reports from other countries. In Sardinia, Italy, samples collected from domestic and wild pigs between 2020 and 2022 showed that PCV2d had largely replaced PCV2b, following a pattern similar to that described in North America and Asia [10]. By contrast, surveillance data from Jilin province, China, collected between 2016 and 2021, showed PCV2b as the most frequent genotype, followed by PCV2e and PCV2d, with no evident recombination signals [50]. This difference may reflect regional variation in the timing and progression of PCV2 genotype replacement.

The PCV2d genotype stands out for its more recent emergence and rapid expansion, especially in North America and China since the early 2010s. It is estimated that PCV2d emerged approximately 20 years ago and it is progressively displacing PCV2b as the predominant genotype in several regions, representing a significant evolutionary shift in the virus’s epidemiology [7]. From a historical perspective, the genotype succession patterns identified in this study coincide with those identified in retrospective analyses in Europe and North America. In Spain, in retrospective analyses covering 1985 to 2008, a genotype shift from PCV2a to PCV2b was reported, coinciding with the occurrence of clinical PMWS outbreaks [51]. Similarly, in the United States, the emergence of PCV2b was recorded between 2005 and 2006. Subsequently, in 2012, a mutant PCV2b variant was identified, showing high genetic similarity to virulent strains detected in China [52], and was linked to clinical outbreaks despite vaccination. These findings support the hypothesis that mutations and natural selection contribute to PCV2 evolution and may influence viral fitness and antigenic variation. However, the specific mechanisms underlying the expansion of PCV2d, including any role of immune-mediated selection, require direct experimental and epidemiological evaluation.

PCV2d is characterized by a mutation that introduces an additional lysine at position 234 of the capsid protein (234-K), which has been associated with higher viral load and an increased frequency of PCVAD-compatible lesions in experimental models [47]. This same change was observed in 50 out of the 51 PCV2d sequences obtained in this study; only one sequence retained an asparagine at the relevant position. The high proportion of sequences classified as PCV2d (~73%) further confirms its predominance and underscores the need to update surveillance, diagnostic, and disease control strategies in the region.

The B-cell epitope prediction using JX535296.1 (PCV2d) as the reference sequence [47] identified nine capsid regions with BepiPred scores suggestive of potential immunological recognition. Analysis of the amino acid variability among the 70 sequences from this study revealed that Epitope 7 (Ep7) was the most frequently mutated epitope, despite exhibiting low per-site divergence (7.14%), indicating that it is the epitope with the greatest qualitative diversity across both PCV2a and PCV2d sequences. Notably, Ep7 has previously been shown to be immunogenic in experimental assays [48]. In contrast, Ep1, Ep2, and Ep5 were largely conserved, with only isolated changes, while Ep4 and Ep6 showed variability primarily among PCV2a sequences and Ep8 and Ep9 were mostly conserved among PCV2d sequences but variable in some PCV2a sequences. The high frequency of amino acid changes in Ep7 indicates that this region is one of the most variable predicted epitopes in the dataset. However, whether this variability affects antibody recognition or contributes to immune escape requires functional validation through immunological assays. The detection of clear recombination signals in seven sequences further indicates active circulation of recombinant variants in the region, which could influence epitope variability and antigenic patterns. Overall, these findings highlight the importance of continued surveillance of both sequence and epitope diversity to anticipate shifts in viral adaptation and support future evaluations of antigenic diversity and vaccine performance.

Analysis of recombination breakpoints in the seven recombinant sequences revealed that several of these events overlap with predicted B-cell epitopes. Notably, Epitope 7 (Ep7), the epitope with the highest frequency of sequence variation, was affected by breakpoints in all seven recombinant events, suggesting that recombination may contribute to its observed diversity. Ep4 and Ep6, which showed moderate to high variability primarily among PCV2a sequences, were involved in four recombinant events: three in PCV2a and one in PCV2d. In contrast, highly conserved epitopes such as Ep1, Ep2, and Ep5 were largely unaffected by recombination, highlighting the preservation of key structural or functional regions despite genetic exchange. These observations suggest that recombination may contribute to sequence diversity in predicted epitope regions. Nevertheless, the antigenic and immunological relevance of these changes cannot be determined from sequence analysis alone and should be evaluated through functional studies.

Recombination, particularly in ORF2 encoding the capsid, is an important evolutionary mechanism that can alter viral antigenicity [53]. Although a functional characterization of these variants was not performed, their presence may have antigenic relevance because most current vaccine formulations are based on PCV2a. These findings support the need to evaluate vaccine performance under local field conditions and to continue monitoring the genetic and antigenic diversity of circulating PCV2 strains.

The recombinant sequences and their putative parental strains are located within a major production and movement corridor that connects the Guadalajara metropolitan area with Altos Sur and Altos Norte. This area includes the highest-swine-density region of Jalisco (B1, B2, and B3), where the movement of animals (including to slaughterhouses), supplies, and technical personnel is common.

High-density production systems can facilitate viral recombination by increasing the probability of co-circulation and coinfection with different PCV2 genotypes within and between farms. Under such conditions, individual animals may harbor multiple viral strains simultaneously, providing opportunities for genetic exchange during replication. Therefore, intensive production regions with frequent animal and personnel movements may represent ecological niches in which the emergence and dissemination of recombinant PCV2 variants are favored.

Previous studies in Jalisco have identified deficiencies in farm biosecurity as relevant risk factors for PCV2 presence, including limited control at farm entrances, inadequate vehicle disinfection, and frequent movement of veterinary service personnel [19]. Within this epidemiological context, the emergence of recombinant variants is biologically plausible. Notably, recombination events 6 and 7 were detected in samples from pigs showing respiratory signs and/or wasting or poor growth.

Natural recombinant PCV2 strains involving different genotypes have recently been reported in India, where recombination between PCV2a and PCV2b was identified within the *cap* gene [1]. In the present study, several recombinant sequences were also detected with strong statistical support (*p* < 0.001), and putative parental strains were mainly assigned to PCV2a and PCV2d. Recombination breakpoints were located across the coding region, and several events were supported by at least five methods, including RDP, GENECONV, MaxChi, Chimaera, and 3Seq. Although BootScan, PhylPro, and LARD did not identify significant signals, the use of multiple complementary methods strengthened the analysis. These findings support continued monitoring of PCV2 genetic evolution in regions where different genotypes co-circulate.

The two extended ORF2 variants identified in this study contained 24 additional nucleotides, resulting in a predicted capsid protein of 238 amino acids. These sequences were analyzed separately because their different ORF2 lengths could bias comparative analyses based on the standard ORF2 alignment. Similar ORF2 extensions have been reported in unusual PCV2 variants, with insertions in the carboxyl-terminal region of the capsid protein, producing a 238-amino-acid capsid instead of the typical 233–234 amino acids described for classical genotypes [54,55]. These findings support the need for whole-genome characterization to better determine the evolutionary placement and biological relevance of such variants.

Based on the observed evidence, it is essential that the study of intra-genotypic variability of PCV2d be deepened to better understand its evolution and epidemiological implications. Moreover, it is vital to assess the representative of the obtained sequences regarding viral diversity in other regions of the country to construct a broader and more contextualized view.

Finally, the results justify studies to evaluate vaccine efficacy under local conditions, especially given the persistent circulation of variants with amino acid changes in predicted epitope regions. Therefore, it is recommended that future research includes more in-depth genetic and antigenic analyses of recombinant variants, as well as experimental evaluations under field conditions, to determine their clinical and epidemiological impact. This knowledge will enable the optimization of PCV2 control and prevention strategies, ensuring better health protection for the swine population in the region.

## 5. Conclusions

The findings show that PCV2d is predominant in Jalisco, Mexico, according to the ORF2 sequences analyzed, in agreement with the genotype replacement pattern reported globally in recent years. The coexistence of PCV2a with PCV2d, together with the detection of recombinant variants and sequences exhibiting unusual ORF2 extensions, indicates ongoing genetic diversification of PCV2 in the region. These patterns may be influenced by multiple epidemiological factors, including animal movement and the co-circulation of distinct genotypes; however, the role of vaccination pressure or immune-mediated selection requires direct evaluation. These findings emphasize the need to strengthen molecular and antigenic PCV2 surveillance, evaluate vaccination strategies under local conditions, and conduct functional studies to evaluate the clinical and epidemiological impact of emerging variants. A deeper understanding of the genetic and antigenic diversity of PCV2 is essential for designing effective control measures that ensure the sanitary sustainability of pig farming in Jalisco and throughout the country.

## Figures and Tables

**Table 1:** List of the 70 PCV2 sequences obtained in this study, including their geographic and epidemiological origin, genotype, Ct value, and GenBank accession number.

Region	Production System	Farm Type	Pig Age Range	Health Condition	Genotype	Ct Value	GenBank Accession
A	FF	S-I	Weaning to 10 weeks	AH	d	26.15	PV235537
A	FF	S-I	Weaning to 10 weeks	RP, W	d	28.25	PV235567
A	FF	S-I	Weaning to 10 weeks	RP, W	d	26.22	PV235571
A	FF	S-I	11 to 14 weeks	AH	d	27.12	PV235562
A	FF	S-I	11 to 14 weeks	RP	d	26.53	PV235568
A	FF	S-I	11 to 14 weeks	RP	d	29.45	PV235569
A	FF	S-I	11 to 14 weeks	AH	d	28.23	PV235590
A	FF	S-I	15 to 18 weeks	AH	a	29.34	PV235587
A	FF	S-I	15 to 18 weeks	AH	d	29.54	PV235563
A	FF	S-I	15 to 18 weeks	AH	d	29.65	PV235564
A	FF	S-I	15 to 18 weeks	RP	d	28.24	PV235566
A	FF	S-I	15 to 18 weeks	RP	d	29.82	PV235572
A	FF	S-I	19 to 22 weeks	AH	d	29.73	PV235576
A	FF	I	Birth to weaning	AH	d	24.3	PV235549
A	FF	I	Weaning to 10 weeks	RP, W	d	23.56	PV235570
A	FF	I	11 to 14 weeks	AH	d	27.41	PV235543
A	FF	I	11 to 14 weeks	AH	d	30	PV235544
A	FF	I	11 to 14 weeks	AH	d	24.52	PV235550
A	FF	I	15 to 18 weeks	AH	d	26.83	PV235545
A	FF	I	15 to 18 weeks	RP	d	29.57	PV235565
A	FF	I	15 to 18 weeks	AH	d	29.52	PV235588
A	FF	I	15 to 18 weeks	AH	d	29.46	PV235589
A	FF	I	19 to 22 weeks	AH	d	29.45	PV235546
B1	FF	S-I	Birth to weaning	AH	a	27.88	PV235547
B1	FF	S-I	Birth to weaning	AH	a	25.16	PV235555
B1	FF	S-I	Birth to weaning	AH	a	29.92	PV235581
B1	FF	S-I	Weaning to 10 weeks	AH	a	25.61	PV235548
B1	FF	S-I	Weaning to 10 weeks	AH	a	25.51	PV235556
B1	FF	S-I	Weaning to 10 weeks	AH	a	26.08	PV235557
B1	FF	S-I	Weaning to 10 weeks	AH	a	27.15	PV235582
B1	FF	S-I	Weaning to 10 weeks	AH	a	29.23	PV235583
B1	FF	S-I	Weaning to 10 weeks	RP, W	d	29.97	PV235573
B1	FF	S-I	11 to 14 weeks	AH	a	29.03	PV235528
B1	FF	S-I	11 to 14 weeks	RP	a	18.08	PV235574
B1	FF	S-I	11 to 14 weeks	RP	a	29.18	PV235575
B1	FF	S-I	11 to 14 weeks	AH	a	29.18	PV235584
B1	FF	S-I	11 to 14 weeks	AH	a	29.38	PV235585
B1	FF	S-I	11 to 14 weeks	AH	d	29.17	PV235586
B1	FF	S-I	15 to 18 weeks	AH	d	29.19	PV235535
B1	1-MS	S-I	Weaning to 10 weeks	AH	a	24.31	PV235559
B1	1-MS	S-I	Weaning to 10 weeks	AH	d	27.56	PV235558
B1	3-MS	I	11 to 14 weeks	AH	a	25.05	PV235533
B1	3-MS	I	11 to 14 weeks	AH	a	28.07	PV235534
B2	FF	S-I	Weaning to 10 weeks	AH	d	29.58	PV235578
B2	FF	S-I	15 to 18 weeks	AH	a	20.03	PV235579
B2	FF	S-I	19 to 22 weeks	AH	a	29.4	PV235580
B2	FF	I	Birth to weaning	AH	d	19.2	PV235551
B2	FF	I	Birth to weaning	AH	d	27.99	PV235552
B2	FF	I	Weaning to 10 weeks	AH	d	26.9	PV235553
B2	FF	I	Weaning to 10 weeks	AH	d	27.61	PV235521
B2	FF	I	11 to 14 weeks	AH	d	29.62	PV235523
B2	FF	I	11 to 14 weeks	AH	d	24.96	PV235524
B2	FF	I	11 to 14 weeks	AH	d	23.45	PV235529
B2	FF	I	11 to 14 weeks	AH	d	26.05	PV235530
B2	FF	I	11 to 14 weeks	AH	d	26.37	PV235541
B2	FF	I	11 to 14 weeks	AH	d	26.03	PV235554
B2	FF	I	15 to 18 weeks	AH	d	26.14	PV235522
B2	FF	I	15 to 18 weeks	AH	d	29.62	PV235531
B2	FF	I	19 to 22 weeks	AH	d	27.21	PV235532
B2	FF	I	19 to 22 weeks	AH	d	28.88	PV235538
B2	FF	I	19 to 22 weeks	AH	d	29.21	PV235542
B2	3-MS	I	11 to 14 weeks	AH	d	29.29	PV235560
B2	3-MS	I	15 to 18 weeks	AH	d	29.6	PV235561
B3	FF	S-I	11 to 14 weeks	AH	d	26.44	PV235527
B3	FF	S-I	11 to 14 weeks	AH	d	23.92	* PV235539
B3	FF	S-I	11 to 14 weeks	AH	d	25.99	* PV235540
B3	FF	S-I	11 to 14 weeks	AH	d	29.78	PV235577
B3	FF	I	15 to 18 weeks	AH	d	29.95	PV235525
B3	FF	I	19 to 22 weeks	AH	d	28.47	PV235526
B3	3-MS	I	15 to 18 weeks	AH	d	26.83	* PV235536

FF: Farrow-to-Finish Farm; 1-MS: 1 Site, Multisite Farms; 3-MS: 3 Sites, Multisite Farms I: Intensive Farm; S-I: Semi-Intensive Farm; AH: apparently healthy; RP: respiratory problems; W: wasting or poor growth; * sequence obtained from a farm that reported not using vaccination for PCV2 control. Ct: cycle threshold value obtained during PCV2 qPCR screening.

**Table 3:** Recombinant PCV2 sequences identified in this study. The table lists the recombinant genotype, inferred parental sequences, estimated recombination breakpoints (nucleotide positions), and statistical support (*p*-values) from different detection methods.

Recombinant Sequences (Genotype)		Parental Sequences (Genotype)		Breakpoint		Detection Methods					
		Minor	Major	Begin	End	RDP	GENECONV	Maxchi	Chimaera	SiSscan	3Seq
1	PV235528 (PCV2a)	PV235525 * (PCV2d)	PV235556 (PCV2a)	621	700	5.09 × 10^−16^	1.08 × 10^−15^	4.41 × 10^−12^	3.59 × 10^−9^	NS	6.21 × 10^−23^
2	PV235525 (PCV2d)	PV235523 (PCV2d)	PV235575 * (PCV2a)	442	178	1.74 × 10^−7^	5.92 × 10^−9^	6.58 × 10^−13^	2.82 × 10^−11^	4.33 × 10^−15^	4.30 × 10^−13^
3	PV235559 (PCV2a)	PV235548 (PCV2a)	PV235523 (PCV2d)	642	270	NS	2.13 × 10^−5^	9.49 × 10^−13^	2.01 × 10^−12^	1.18 × 10^−13^	3.74 × 10^−20^
4	PV235584 (PCV2a)	PV235573 (PCV2d)	PV235533 (PCV2a)	668	314	5.42 × 10^−3^	2.01 × 10^−4^	9.17 × 10^−11^	2.36 × 10^−8^	NS	2.58 × 10^−13^
5	PV235555 (PCV2a)	PV235523 (PCV2d)	PV235548 (PCV2a)	346	563	4.04 × 10^−10^	1.56 × 10^−7^	2.14 × 10^−7^	4.04 × 10^−9^	9.66 × 10^−10^	1.18 × 10^−10^
6	PV235573 (PCV2d)	PV235533 (PCV2a)	PV235523 (PCV2d)	690	226	NS	8.35 × 10^−7^	3.08 × 10^−8^	8.23 × 10^−7^	NS	7.46 × 10^−10^
7	PV235575 (PCV2a)	PV235523 (PCV2d)	PV235579 (PCV2a)	226	284	9.77 × 10^−5^	6.67 × 10^−4^	7.85 × 10^−5^	2.15 × 10^−3^	2.30 × 10^−4^	5.48 × 10^−5^

* Sequence identified as a donor of genetic material in a recombination event based on phylogenetic and statistical evidence but could not be reliably classified as major or minor parent by the applied statistical methods. The analysis included Bootscan, PhylPro, and LARD, all of which did not detect significant evidence of recombination.

## Data Availability

Data from the studied farms are not available due to privacy restrictions.

## Abbreviations

The following abbreviations are used in this manuscript:

A, B1, B2, B3	Designations of pig density regions in Jalisco
Ct	Cycle threshold
DNA	Deoxyribonucleic acid
FF	Farrow-to-Finish commercial farm
FW	Forward primer
INB	National Institute of Biodiversity
INIFAP	Instituto Nacional de Investigaciones Forestales, Agrícolas y Pecuarias
Kb	Kilobase
MS	Multisite farms
ORF	Open Reading Frame
PCR	Polymerase Chain Reaction
PCV2	Porcine Circovirus Type 2
PCVADs	Porcine circovirus-associated diseases
PDNS	Porcine dermatitis and nephropathy syndrome
PMWS	Postweaning multisystemic wasting syndrome
PRDC	Porcine respiratory disease complex
qPCR	Quantitative PCR
R	Statistical programming language and environment
RStudio	Software for statistical analysis based on R
RV	Reverse primer
SIACON	Sistema de Información Agroalimentaria de Consulta
ssDNA	Circular single-stranded DNA
TAE	Buffer Tris-acetate-ethylenediaminetetraacetic acid
UNAM	National Autonomous University of Mexico
URPJ	Unión Regional de Porcicultores de Jalisco
